# Draft Genome Sequence of Pectobacterium brasiliense Isolated from Potato in South Africa

**DOI:** 10.1128/mra.00763-22

**Published:** 2023-03-22

**Authors:** Molemi Evelyn Rauwane, Nokuthula Peace Mchunu, Rian Pierneef, Valery Mamogwasha Moloto, Teresa Goszczynska, Phumzile Sibisi, Khayalethu Ntushelo

**Affiliations:** a Department of Agriculture and Animal Health, University of South Africa, Florida, South Africa; b Agricultural Research Council – Biotechnology Platform, Onderstepoort, South Africa; c Agricultural Research Council – Plant Health and Protection Research, Roodeplaat, South Africa; d National Research Foundation, Pretoria, South Africa; University of Maryland School of Medicine

## Abstract

This study reports a draft genome of a phytopathogenic bacterium, Pectobacterium brasiliense, isolated from potato in South Africa. The total reported length of the genome is 4,897,858 bp, contained in 172 contigs with 4,378 genes. The GC content of the genome is 51.6%.

## ANNOUNCEMENT

Pectobacterium brasiliense (1) (strain BD163) was isolated from a rotting potato tuber in Roodeplaat, Tshwane, South Africa. This strain was chosen for whole-genome sequencing because it has been earmarked for upcoming experiments on plant-microbe interactions, and therefore, its genome must be fully understood. A disease lesion from the potato tuber was cut into a sterile petri dish and then macerated into 1,000 μL of sterile distilled water. The macerate was left in a laminar flow for 15 min for the bacteria to disperse, and aliquots of 100 μL were streak plated on Luria-Bertani agar. A single colony was picked and grown on tryptone extract agar, and it was further transferred to Luria-Bertani broth and incubated overnight on a rotary shaker, shaking at 150 rpm at 28°C (2). Preliminary identification of the strain was done according to the work of Cother and Sivasithamparam (3). From 1 mL of broth, bacterial cells were harvested by centrifugation, and DNA was extracted using the Quick-DNA miniprep Plus kit (Zymo Research, Irvine, CA). Libraries of genomic DNA were prepared using the TruSeq Nano DNA library preparation kit, and then sequencing was done using the Illumina MiSeq sequencing platform to produce paired-end sequences of 300 bp. Quality control of the raw data including adapter removal was done using BBDuk (v.37.90) (4). SPAdes v.3.15.3 (5) was used to create a *de novo* assembly of *P. brasiliense*. Only contigs larger than 500 bp were retained for further analysis. Assembly quality assessment was done using QUAST v5.0.2 (6), genome quality was inspected with CheckM v.1.1.3 (7), and initial classification was performed using GTDB-Tk v.0.3.2 (8). Genome annotation was done using PGAP v6.0 (9). All software programs were run with default parameters. The genome assembly contained 172 contig sequences ranging from 500 bp to 1,658,186 bp in length (Table 1). A total of 4,897,858 bp was assembled, covering the full genome containing 4,378 genes and 70 tRNA genes. The bacterial strain was confirmed to be *P. brasiliense* because its genome sequence was 98.38% similar to the genome sequence of the reference strain of *P. brasiliense*, LMG 21371 (NCBI accession no. GCA_000754695.1).

**TABLE 1:** General features of *Pectobacterium brasiliense* genome

Feature	Value
Genome size	4,897,858 bp
Genome representation	Full
No. of contigs	172
*N*_50_ contig	824,806 bp
No. of predicted genes	4,378
GC content	51.6%
No. of tRNA genes	70

### Data availability.

The draft genome sequence of *P. brasiliense* BD163 is found in the National Center for Biotechnology Information (nih.gov) under BioProject no. PRJNA798891, and the raw reads are under accession no. JAKNTB000000000 (*Pectobacterium brasiliense* strain BD163, whole-genome shotgun sequence—Nucleotide—NCBI [nih.gov]). The BioSample number is SAMN25132833, and the GenBank assembly accession number is GCA_022172285.1.
